# Fundamental Discoveries in Enzymology through Undergraduate Research

**DOI:** 10.1021/acsomega.5c07487

**Published:** 2025-10-08

**Authors:** Sudeep Bhattacharyya, Sanchita Hati

**Affiliations:** Department of Chemistry and Biochemistry, 14747 University of Wisconsin-Eau Claire, Eau Claire, Wisconsin 54702, United States

## Abstract

Undergraduate research has long been recognized as a powerful catalyst for academic and professional development. Many institutions, particularly Primarily Undergraduate Institutions (PUIs), actively promote and support undergraduate research through a variety of initiatives. In this paper, we describe our efforts to engage undergraduate students in authentic and challenging research projects focused on the structure–function-dynamics relationships in multidomain proteins. These projects were pursued through both traditional and course-embedded research models. We highlight the novel contributions of our student researchers to the broader field of enzymology, whose work has led to new insights in the field and resulted in several publications in peer-reviewed journals. Through their research experiences, students not only developed essential laboratory skills and expanded their scientific toolkits but also gained meaningful exposure to real-world problem-solving. These opportunities significantly enhanced their analytical and critical thinking abilities, strengthening their competitiveness for graduate studies and career advancement. Taken together, engaging undergraduates in research helps facilitate discoveries, cultivates a new cohort of future scientists, educators, and a scientifically skilled and empowered workforce.

## Graphical abstract

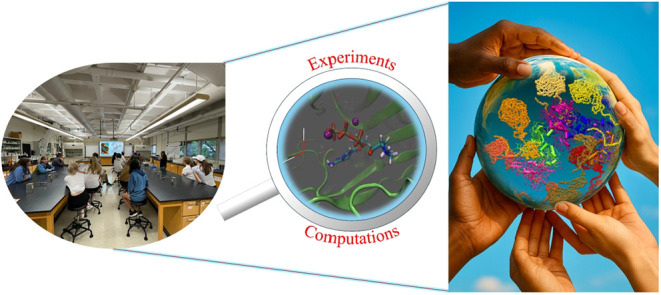

## 1 Introduction

Chemistry is considered the central science for a sustainable future, the science of transformations that fosters critical understanding of our physical world through a rich web of interconnected disciplines.^1,2^ Fields such as biology, physics, mathematics, computational science, informatics, and deep learning each extend a helping hand, offering tools and perspectives that deepen our understanding of chemistry – the study of the fundamental building blocks of matter i.e., the molecules and their transformations. At the heart of this exploration lies curiosity, the driving force that propels us beyond familiar boundaries and into new realms of knowledge. In this context, research becomes a powerful vehicle for discovery. It fosters a mindset that values stepping outside traditional domains in pursuit of truth. Undergraduate research, in particular, has been proven to be the most effective in STEM (Science, Technology, Engineering, and Mathematics) education^3–5^ and provide a meaningful strategy of active learning for chemistry.^6,7^ Grounded in the core discovery process, i.e., observation, hypothesis, experimentation, and conclusion, research cultivates an evidence-based approach to understanding the natural world, embedding this practice deeply in the undergraduate experience.

Located in western Wisconsin, the University of Wisconsin-Eau Claire (UWEC) is one of the Midwest’s leading public universities, offering more than 200 majors, minors, certificates, and graduate programs. UWEC, a Primarily Undergraduate Institution (PUI), has earned national recognition for its longstanding commitment to undergraduate research, consistently ranking among the top institutions in the country in this area. Just in 2025, three UWEC undergraduates were awarded the prestigious Goldwater Scholarship, an honor recognizing outstanding students interested in pursuing research careers in science, engineering, and mathematics. This marks the highest number of Goldwater recipients in a single year for any regional comprehensive public university across six Midwestern states, highlighting a strong commitment to promoting undergraduate research. Within the UWEC campus, the Department of Chemistry and Biochemistry stands out for its five-decade legacy of engaging undergraduates in high-impact research. Faculty-student collaborations have resulted in numerous peer-reviewed publications and conference presentations, making the department a nationally recognized leader in undergraduate chemistry research. This strong research culture has helped UWEC students gain admission to top-tier Ph.D. programs, medical schools, and other professional pathways. According to the NSF’s WebCASPAR database,^8^ UWEC ranks among the top undergraduate institutions in the U.S. for producing future Ph.D. recipients in chemistry, even when compared to the nation’s most prestigious liberal arts colleges. The Department of Chemistry and Biochemistry at UWEC has built a remarkable record of undergraduate education and research excellence because of a unique combination of talented and motivated students, a dedicated and productive faculty, and an administration that prioritizes and supports high-quality academic programs.

In the Department of Chemistry and Biochemistry at UWEC, the Protein Dynamics Lab, led by us (Drs. Bhattacharyya and Hati), investigates the role of protein dynamics in enzymatic functions, while fostering undergraduate research. Since 2006, numerous undergraduates have contributed to exploring the intricate relationship between structure, dynamics, and function in modular enzymes, addressing one of enzymology’s central questions: *What role do intrinsic protein dynamics play in the catalytic functions of modular enzymes?* This interdisciplinary research engages students majoring in a wide range of academic disciplines – artificial intelligence, biochemistry/molecular biology, biology, biomedical engineering, chemistry, computer science, mathematics, and physics. The methodologies employed are equally diverse, encompassing computational modeling, biochemical and molecular biology techniques, spectroscopy, microscopy, statistical analysis, and artificial intelligence. The ongoing research focuses on two major classes of enzyme-catalyzed reactions: (i) electron transfer and (ii) group transfer involving multimeric and multidomain enzymes. Two enzyme families, aminoacyl-tRNA synthetases (AARSs)^9–12^ and quinone reductases (QRs),^13–17^ have been studied in depth with undergraduate collaborators. This review highlights the lab’s approach and strategies to fostering undergraduate research, different computational and experimental projects undertaken in collaboration with undergraduates, and the significant research outcomes achieved at UWEC.

## 2 Methods

Undergraduate research is the hallmark of students’ learning, and the Council of Undergraduate Research (CUR) has been the pioneer in demonstrating that such activity is pivotal to enriching and advancing society.^18,19^ Participation in small-group collaborative research has many advantages in students’ academic growth.^3–5^ It offers students valuable opportunities to apply classroom knowledge to real-world scientific challenges. Such engagement has been shown to enhance educational outcomes, including higher retention and degree completion rates. It also positively influences students’ career trajectories by improving their readiness for advanced study and competitiveness in the job market. Most importantly, undergraduate research experience is often considered essential for admission to graduate and medical schools, alongside strong GPAs and standardized test scores. To broaden access to hands-on scientific inquiry, we have been proactive in involving students in authentic scientific research. For the past several years, efforts have been made to engage undergraduates in authentic research through both traditional and course-embedded research settings.^20^ The following subsections detail strategies for integrating research into undergraduate education, including the design of course-based research projects, approaches to mentoring and training, and initiatives aimed at securing external funding.

### 2.1 Combined Traditional and Course-Embedded Research

Traditional undergraduate research experiences, such as summer internships and independent study during the academic year, are commonly used to engage STEM students in hands-on research. These opportunities typically involve close one-on-one interactions between faculty mentors and mentees, including collaborative brainstorming, experimental work, data analysis, and troubleshooting. However, this traditional form of collaborative undergraduate research often restricts access for many students. In particular, introverted students, first-generation college students, individuals from underrepresented communities, and those outside conventional coursework often hesitate to actively pursue research opportunities due to discomfort with networking or a lack of awareness about available options. Integrating discovery-based research into the classroom, i.e., course-embedded undergraduate research experiences (CURE), offers a promising solution. By extending research opportunities beyond traditional lab settings, faculty can reach a broader and more diverse student population, fostering greater inclusion and engagement within STEM education. Thus, we design authentic research projects delivered through both traditional and course-based models to broaden student engagement in undergraduate research.^20,21^

#### 2.1.1 Faculty-Guided Research in Traditional Lab Settings

In both the experimental (Hati) and computational (Bhattacharyay) laboratories, we recruit three to four undergraduate students each year, primarily those in their sophomore or junior standing. Students are assigned to a research project and typically work either individually or in pairs throughout the academic year and summer. Students typically engage with our research group for at least one year, with many continuing their involvement for up to three years. Projects that are in their early conceptual stages require closer faculty guidance initially, with students gradually assuming greater responsibility as the work progresses. Many projects carry over from year to year, allowing senior students to provide peer-led onboarding and support during the initial training period. Students typically enroll in Independent Studies courses during the academic year, and stipends are arranged through a combination of external grants and internal funding sources.

#### 2.1.2 Faculty-Guided Research in Course-Embedded Settings

Since 2012, the course-embedded research has been integrated into three capstone courses of Chemistry: Biophysical Chemistry, Physical Chemistry, and Biochemistry II. A carefully designed weekly research plan is developed alongside the course syllabus to guide faculty-mentored research projects. For example, in the Biophysical Chemistry course, which includes a dedicated 2 h weekly laboratory session, students engage in discovery-based research using computational chemistry. Students typically work in groups, with each member sharing equal responsibility—from conducting literature reviews to performing experiments and analyzing data. At the end of the semester, each group submits a research writeup. Motivated students are encouraged to enroll in a one-credit Independent Study course the following semester to compile results, present their research at regional and national conferences, and prepare manuscripts for submission to peer-reviewed journals.

### 2.2 Effective Mentoring Practices

One of the core principles of our research group is to foster a collaborative, rather than top-down, approach when working with undergraduate researchers. We view them as true collaborators, with knowledge and ideas flowing in both directions. New lab members are typically trained by more experienced students through a structured, peer-led process. The computational training is conducted by one of the mentors after a student receives an initial training from the system administration staff member of the Blugold Center for High-Performance Computing. Our training is multifaceted, emphasizing not only technical proficiency but also the development of professional, problem-solving, and communication skills.^22^ Student collaborators are actively involved in every phase of their research projects, from conducting experiments and troubleshooting to analyzing data.

Each student follows an individualized development plan designed to support their growth into a competent and confident researcher. We hold regular one-on-one meetings and weekly group sessions. These meetings provide opportunities to discuss research plans, review results, address challenges, design new experiments, and outline goals for the coming week. Students are also encouraged to read and critically evaluate scientific literature and to present their findings during group meetings. At the end of each semester, students submit a journal-style report summarizing their research progress. They also present their work at local, regional, and national conferences. Through this comprehensive training, students gain experience in scientific writing and communication, and most go on to coauthor peer-reviewed publications.

### 2.3 Financial Support for Promoting Sustained Undergraduate Research

Over the years, we have developed a robust and sustainable research program. For conducting research during both the academic year and in summer, we consistently secure external and internal funding to support student stipends, laboratory supplies, and computational resources. Funding is secured through federal agencies, including the National Institutes of Health and the National Science Foundation, as well as local sources. UW–Eau Claire’s Office of Research and Sponsored Programs plays a pivotal role by providing technical assistance for external grant submissions and overseeing internal funding applications. Our research centers on biophysical chemistry with strong relevance to health sciences, integrating computational approaches into biochemical investigations. This interdisciplinary focus has enabled us to successfully obtain funding that advances our research objectives while actively promoting undergraduate participation in cutting-edge scientific inquiry.

#### 2.3.1 Integrating Cyberinfrastructure Resources

One of our key initiatives was securing Major Research Instrumentation (MRI) funding from the National Science Foundation to establish UW–Eau Claire’s first high-performance computing cluster. This transformative resource has enabled STEM students to actively participate in advanced quantum chemical calculations and long-time scale protein simulations, significantly expanding their engagement in computational research, which is one of the essential tools for studying chemistry in the 21st century.^22^

#### 2.3.2 Federal Research Grants

Besides providing answers to fundamental enzymological questions, research projects studied in our lab (*vide infra*) also promise significant insights into health-related aspects. Our lab has received consistent support from the National Institute of Health, enabling us to recruit and mentor six to eight undergraduate researchers throughout the academic year and summer. These opportunities not only ignite our students’ passion for chemistry but also equip them with the skills and experience necessary for advanced studies or entry into the workforce.

#### 2.3.3 Synergistic Integration of Computational Science Across Multiple Disciplines

As part of our broader initiative, we have actively incorporated high-performance computing into the curriculum, particularly during the COVID-19 lockdown. This provided an avenue for cross-disciplinary collaboration and reaped the educational benefit of computational science. In particular, a multidepartmental NSF Research Experiences for Undergraduates (REU) proposal was awarded in 2022 and is being continued. This initiative has reinforced collaborative practices across various STEM fields, especially those reliant on high-performance computing.

#### 2.3.4 Collaboration with Mayo Clinic

UWEC has established a collaborative partnership with the Mayo Clinic Health System. We also explored ways to expand and facilitate undergraduate research via collaborations with physicians at Mayo Clinic, Eau Claire, WI, and Rochester, MN. These collaborations allow students to work alongside academicians and medical professionals on real-world problems such as developing cancer detection methodologies. Through these experiences, undergraduate researchers are encouraged to think creatively, apply classroom knowledge in meaningful ways, and contribute to cutting-edge scientific inquiry.

## 3 Student Learning-Centered Research Program

As structure–function–dynamics relationships in proteins is foundational to biochemistry and biophysics, the majority of our research projects focus on the areas that advance the understanding of this fundamental principle. In collaboration with several motivated undergraduate students each year, we have been successful in creating a student-centered research program to explore four major areas of biophysical chemistry (Figure 1). Area 1 explores the key enzymology question of how protein structure dictates its functional dynamics. Area 2 examines the crowding complications in intracellular environments and their impact on enzymes’ behavior. Area 3 was initiated during the COVID-19 lockdown period, where we investigated the connection between oxidative stress and disease severity. In Area 4, our laboratory collaborates with the Mayo Clinic to develop spectroscopic techniques for disease diagnosis and monitoring.

### 3.1 Protein Structure and Functional Dynamics

A large body of work has been carried out to probe the role of enzyme motions in catalysis. Protein dynamics occur at various time scales and involve a variable range of protein secondary structures.^23–32^ Larger domain motion occurs slowly in the range of milliseconds to a few seconds, while loops move faster in the picosecond to microsecond range. These motions influence the catalytic process by bringing a specific set of side chain atoms involved in the catalysis through a process of reorganization and preorganization of the active site.^25,26^ For small particle transfer reactions (like an electron, proton, or hydrogen atom), the quantum effects^33,34^ are significant – the vibrational motion associated with the bond breaking and forming impacts the catalysis, especially where quantum mechanical tunneling is involved.^35,36^

#### 3.1.1 Dynamics and Hydride Transfer: A Case of Ping-pong Kinetics in Quinone Reductases

One area of interest was focused on the question of ping-pong reactions involved in the Quinone Reductases (QR1 and QR2).^13,15–17^ These groups of enzymes act as detoxifiers of drugs and other substances and are known as phase II enzymes. Chemically speaking, they utilize a two-substrate double displacement reaction through a process known as ping-pong kinetics. The process involves one reductive (Figure 2, step a) half-cycle and one oxidative half-cycle (Figure 2, step c).^37,38^ In the reductive process, a hydride donor substrate transfers a hydride to the cofactor flavin ring. This is followed by a displacement of the product by a hydride acceptor one (Figure 2, step b), which initiates the oxidative half-cycle and removes the hydride, and the cycle continues (Figure 2, step d). Thus, the key mechanism of the ping-pong kinetics involves a hydride-transfer-induced charge separation at the active site. The molecular-level details could enlighten us to the role of protein dynamics.

##### 3.1.1.1 Hydride Transfer Involving Enzyme-Bound Flavin

The flavin ring, also known as the isoalloxazine ring, consists of three fused cyclic ring systems, namely, a dimethylbenzene on the left, a central pyrazine, and an uracil ring on the right (Figure 2). The flavin ring can exist in various electronic states, namely, quinone, semiquinone, and hydroquinone forms. Depending on the pH of the surroundings, these species can also have more than one protonation state. As the back-and-forth hydride-transfer changes flavin redox and protonation–deprotonation states, the charge separation due to hydride transfer must involve significant reorganization of the active site residues, which impacts the Gibbs free energy of the process. Thus, Rauschnot et al. used theoretical methods and atomistic simulations^39^ to determine the redox potentials of the QR2-bound flavin, characterize flavin’s protonation state, and the associated conformational changes of the active site.^39^

The work involved three undergraduates, who gained experience with various computational techniques, such as Linux-based computing, protein visualization and editing, and molecular dynamics (MD) simulations using hybrid quantum mechanical/molecular mechanical (QM/MM) potentials. Students learned how to think critically and rationally to explain the change in flavin’s redox potential due to interactions with the side chains in the enzyme’s active site.

**Figure 1:**
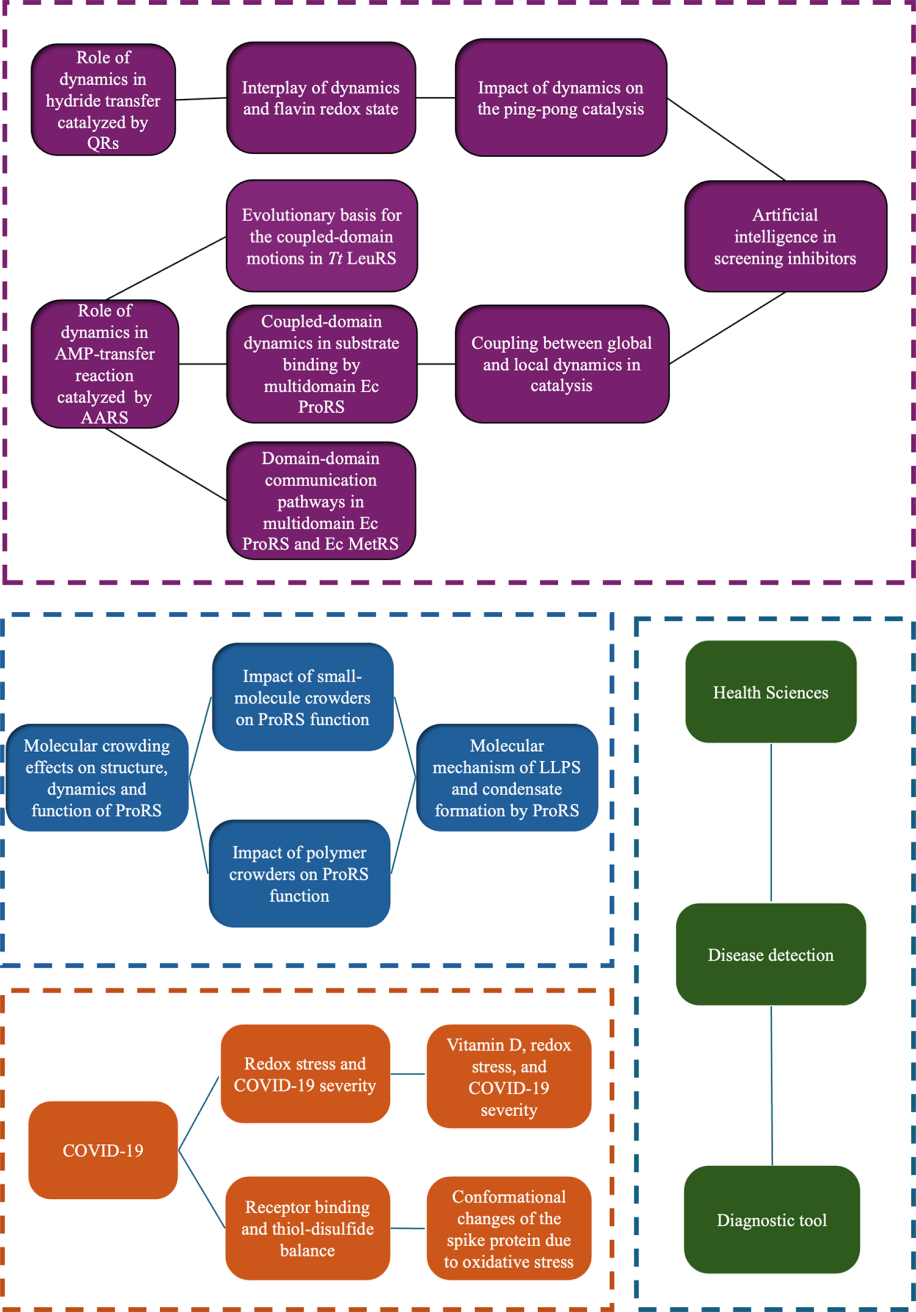
Four broadly defined areas with the subprojects pursued in the Protein Dynamics group at UW-Eau Claire: area 1 in purple, intrinsic dynamics and enzymatic function; area 2 in blue, crowding, confinement, and condensate formation; area 3 in orange, COVID-19; area 4 in green, health sciences.

**Figure 2:**
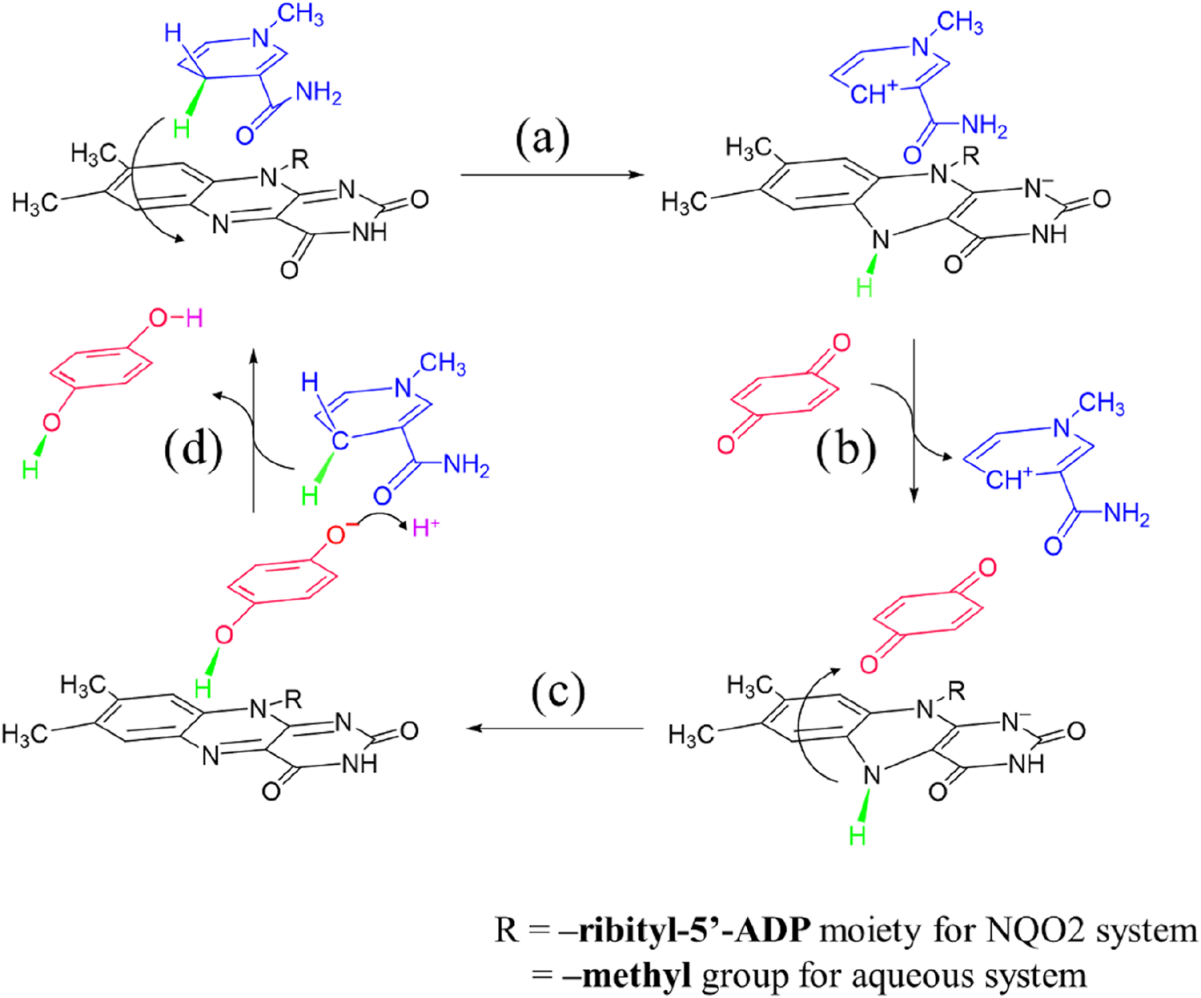
“ping-pong” catalysis in quinone reductase 2, where hydride donor and acceptor molecules engage in a substrate shuttling dynamics (adapted from ref 38). The catalytic cycle consists of two redox half-cycles, each involving a catalytic hydride transfer step (step a or c) followed by a product displacement step (step b or d).

##### 3.1.1.2 Hydride-Transfer-Induced Changes in Collective Dynamics

The interplay of the redox process and active site dynamics was investigated as a follow-up. The impact of flavin’s redox state on the collective dynamics was explored. Critical evidence was obtained by neutralizing the charges of the selected charged residues and examining their impact simultaneously on the stabilization of the quantum chemically treated subsystem and the projection of the principal component of motions. Using the analysis, Mueller et al. demonstrated that the active site stabilizing the reduced flavosemiquinone exhibited distinctly different dynamics than the one bound to the neutral oxidized flavin. Closer scrutiny further revealed that the observed dynamic changes are correlated with the energetics and are influenced by the altered electrostatic interactions between the flavin ring and the active site residues.^37^

Students participating in this particular study became exposed to several theoretical techniques of computational enzymology, such as free energy perturbation, collective dynamics analysis, and charge-neutralization calculation (Table 1). In addition, they gained valuable expertise in visualization of principal components of protein motions.^37^

##### 3.1.1.3 π–π Stacking Interactions with Flavin and Aromatic Molecules

As the catalysis of quinone reductases predominantly involves π–π interactions between the flavin ring and the substrate or product, our effort has been to study the nature of interactions between the flavin ring and several aromatic systems with delocalized π electrons.^40^ Using benchmark calculations, the study demonstrated that the nature of interactions is certainly of dispersion type and can be efficiently modeled by M06–2X functional^41^ and density-functional tight-binding^42^ that can work well with hybrid quantum mechanical/molecular mechanical potentials. The study by Bresnahan et al. revealed that the M06–2X hybrid functional most accurately models π–π interactions, which are primarily governed by medium-range electron correlation effects. Furthermore, the study demonstrated that, unlike the thermodynamics of the flavin’s redox change, the kinetics of the hydride transfer reaction from *N*-methyl nicotinamide to flavin was strongly influenced by the stacking interactions.^41^

This work provided a great opportunity for students to learn about the physical basis of the medium-range electron correlation effects in the context of stacking interactions between molecular systems with delocalized π electrons (Table 1). Dispersion interactions are caused by instant multipole-induced multipole interactions, which are due to the rhythmic correlated fluctuation of the electron density – a manifestation of the medium-range electron–electron correlation effect. Students were able to study these interactions by plotting the attractive region of the potential energy and the distance of separation between the ring systems. The presence of dispersion-type interactions was supported by their characteristic dependence on the inverse sixth power of the distance of intermolecular separation.

##### 3.1.1.4 Hydride Transfer from Flavin to Substituted Quinones

Quinone reductases catalyze hydride transfers from flavin to quinone, which necessitates π–π stacking of the quinones onto flavin ring. As the binding interaction is the key component of the activated complex, the hydride transfer kinetics between lumiflavin and a number of substituted quinones were probed in a subsequent study using density functional theory.^43^ The work demonstrated that for quinones with electron-withdrawing substituents, the activation barrier reduces significantly. In addition, the transition state appears to undergo a shift toward the reactants side because of the electron density being withdrawn from the quinone group.

Through this study, students learned how the fundamentals of thermodynamics and kinetics are linked, and while working with the hydride transfer reaction between flavin and substituted quinones, they experienced firsthand how the Marcus law-predicted quadratic relationship between the activation barrier and the driving forces. As part of the project guideline, students received specific instructions to analyze results, create reports, and present them accordingly. This project was embedded in a physical chemistry course; a total of 17 students along with a research student coauthored the article.^43^

##### 3.1.1.5 Substrate Shuttling in Ping-Pong Catalysis

The hydride transfer reactions in QR2 were probed by theoretical means. Using QM/MM method, the response of the enzyme active site matrix was probed by computing the free energies of the two catalytic hydride transfer steps and two product displacement steps. This study provided a clear picture of the oscillatory behavior of the active site that was portrayed through a complex interplay of solvation, protonation, and protein matrix-induced polarization that acts as the driving force behind the thermodynamic wheel of the “ping-pong” kinetics. The detailed analysis of the movement of the charged amino acids exhibited a cyclic change in the active site polarization that governs the oscillatory dynamics in this enzyme.^38^

Students learned to perform a series of MD simulations to model enzyme catalysis. They understood the molecular-level details of how dynamics plays a role in a multisubstrate enzymatic reaction. This study helped students to witness how the charge separation caused by the transfer of hydride is responded to by the protein matrix using dynamics and reorganization of the active site electrostatics.

**Table 1:** Computational and Experimental Tools Utilized by Students in Protein Chemistry Research

**research areas**	**methods and tools**
**protein structure and functional dynamics**	*experimental*
	protein purifications, site-directed mutagenesis, enzyme kinetics, spectroscopy
	*computational*
	homology modeling, atomistic model building of solvated proteins, QM/MM simulation, principal component analysis, normal-mode analysis
**crowding, confinement, and condensation**	*experimental*
	fluorescence spectroscopy, atomic force microscopy, dynamic light scattering, confocal microscopy, calorimetry
	*computational*
	electronic structure calculations, atomistic model building of solvated protein-crowder system, molecular dynamics simulation, intermolecular interaction energy calculations
**oxidative stress and conformational changes of spike protein**	*literature review*
	redox processes in biochemical pathways, key enzymes involved, and the underlying causes of redox stress.
	Receptors involved in coronavirus entry and its redox state
	*computational*
	atomistic model building of solvated protein system, molecular dynamics simulation, protein–protein binding affinity calculations
**spectroscopic tools for disease diagnostics**	*spectroscopy*
	biological sample preparation, nanoparticles handling, spectroscopic analysis

#### 3.1.2 Dynamics and Group Transfer: A Case of AMP-transfer Catalyzed by Aminoacyl-tRNA Synthetases

AARSs play a pivotal role in protein biosynthesis and maintaining accuracy during the translation of the genetic code. These ″housekeeping″ enzymes catalyze the aminoacylation of tRNAs with cognate amino acids in a two-step reaction to provide charged tRNA for protein synthesis. AARSs consist of multiple domains.^44^ The central catalytic core (Figure 3) is responsible for the activation of an amino acid in the presence of ATP to form an enzyme-bound aminoacyl-adenylate (AA-AMP; eq 1) and transfer of that activated amino acid to the 3′- end of its cognate tRNA (eq 2). The eq 1. could be considered as transfer of an AMP group to an amino acid.

$$\mathrm{AARS}+\mathrm{AA}+\mathrm{ATP}\to \mathrm{AARS}\cdot \left(\mathrm{AA}‐\mathrm{AMP}\right)+{\mathrm{PP}}_{i}$$ 1

AARS·(AA‐AMP)+tRNA→AA‐tRNA+AARS+AMP 2

The anticodon binding domain possesses recognition elements for tRNA. In the course of evolution, additional domains were either appended or inserted into the two-domain structure to enhance catalytic efficiency and confer tRNA selection.^10,12^ For example, some synthetases have evolved editing mechanisms; they possess a separate editing domain for hydrolyzing misactivated amino acids *(pretransfer editing*) and misacylated tRNAs (*post-transfer editing)*.^12^

For AARSs, we sought to find the answer to a fundamental question, which still occupies the central dogma of enzymology – why are enzymes so large, while the chemistry occurs in the small active site pocket comprising a handful of atoms of a small number of side chains? With this query, one of our initial projects was to understand the role of distant domains in catalysis and how different domains communicate in multidomain AARSs (Figure 3) that carry out several tasks in a synchronized manner as the aminoacylation of tRNA involves a series of events that includes selection of the correct amino acid and its activation in the presence of ATP, binding of the cognate tRNA, transfer of the activated amino acid onto the cognate tRNA, release of the aminoacylated tRNA from the enzyme active site, and in some cases, proofreading.^10,12^

Biochemical and structural studies have demonstrated the existence of allostery in AARSs, where a perturbation far from the catalytic domain has a significant impact on the enzyme function. For example, point mutations in a distant domain in *E. coli* prolyl-tRNA synthetase (Ec ProRS) (Figure 3), *Thermus thermophilus* leucyl-tRNA synthetase (Tt LeuRS), and *E. coli* methionyl-tRNA synthetase (Ec MetRS) have a significant impact on substrate binding and catalysis.^46–48^ Additionally, the covalent connectivity between domains is also found to be crucial for AARSs’ function. All these observations indicated that distant functional sites of an AARS are energetically coupled. However, both the mechanism of interdomain communication and the role of intrinsic global and local dynamics on ligand binding affinity and/or catalytic rate were not fully understood, which led to detailed investigations in our group.

Enzymologists have demonstrated two key points: first, that interdomain communication is conserved through evolution,^31^ and second, that enzymes primarily rely on intrinsic motions to facilitate these communications.^32^ Building on this foundation, our undergraduate-led research group investigated these interdomain interactions and mapped communication pathways, revealing that residues involved in signaling between functional sites are either evolutionarily conserved or have coevolved to preserve functional dynamics. The coupling of domain dynamics appears to support localized motions essential for maintaining enzymatic catalysis. In collaboration with several undergraduate researchers, we explored the relationship between structure, dynamics, and function in AARSs as described here.

##### 3.1.2.1 Cooperative Domain Dynamics in Tt LeuRS

For Class I LeuRS, which catalyzes the covalent attachment of leucine to tRNA^Leu^, conformational changes in various structural elements, as revealed by X-ray crystallography, suggest that large-scale flexibility and domain movements play a critical role in both aminoacylation and editing reactions. By employing normal-mode analysis and statistical coupling analysis, three undergraduate researchers collaborated in identifying coevolved and conserved residues in multidomain Tt LeuRS, which are crucial for communication between distantly located domains through the coordinated movements of structural elements. Notably, long-range communication between these domains is mediated by coupled-domain dynamics. This study represents the first identification of coupled-domain motions among evolutionarily relevant residues in this enzyme family.^47^

Undergraduates working on this project learned to perform normal-mode analyses and gained an understanding of how low-frequency normal modes represent large-scale, collective motions in proteins, motions that are often biologically relevant. Additionally, through the application of statistical coupling analysis, they explored the differences between conserved and coevolved residues, and how these residues contribute to maintaining both the structural integrity and dynamic behavior necessary for protein function.

##### 3.1.2.2 Role of Coupled Dynamics in Substrate Binding

The two distant domains of Ec ProRS, namely the catalytic (aminoacylation) and the editing or insertion (INS) domains, which are responsible for amino acid activation and post-transfer editing, respectively, have been observed to depend on each other in terms of their catalytic activities. Moreover, complete deletion of the INS domain of Ec ProRS has a severe effect on catalysis; the amino acid activation efficiency of the deletion mutant (ΔINS) was reduced by 1200-fold, and a separately cloned and purified INS domain in *trans* failed to stimulate the amino acid activation efficiency of the ΔINS construct.^49^ These results demonstrated that the catalytic and editing domains, although situated ∼30 Å apart, do indeed communicate with each other during catalysis.

**Figure 3:**
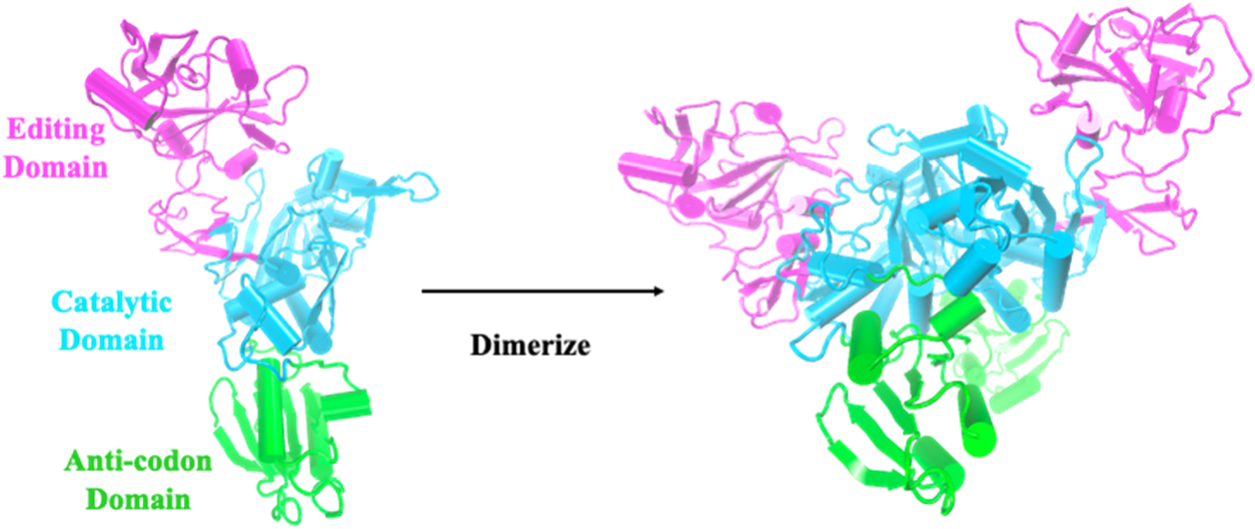
Three domains of *Escherichia coli* ProRS.^45^

By employing computational (MD and PCA) and experimental (site-directed mutagenesis and kinetic studies) approaches, undergraduate researchers, along with their PIs established that the global dynamics of the INS are coupled with the local dynamics of the catalytically important proline-binding loop (PBL),^45^ which undergoes a large-scale (movement of ∼8 Å) conformational transition from ″open″ to ″closed″ state upon substrate binding (Figure 4).

**Figure 4:**
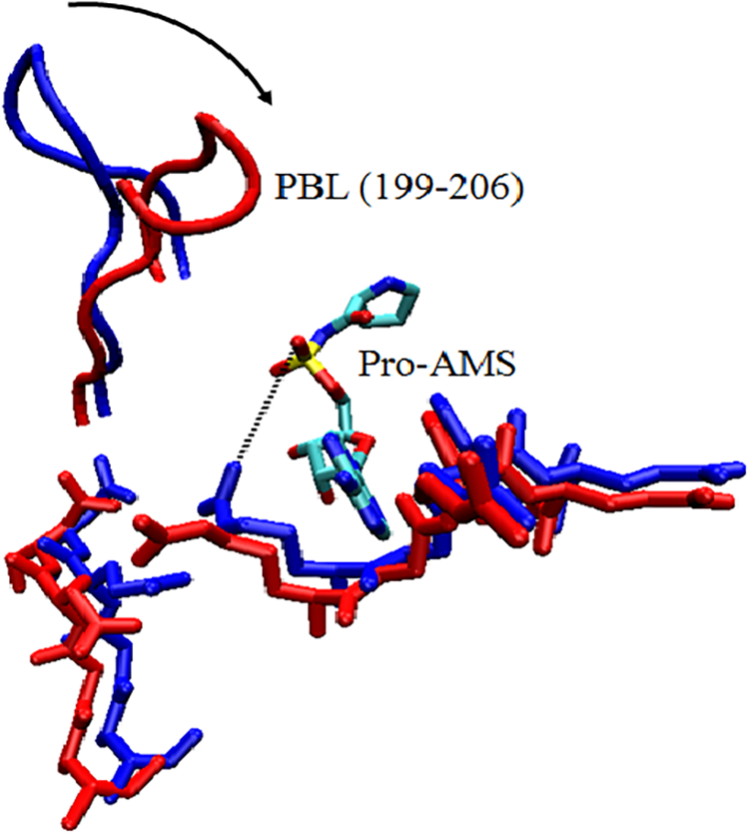
Conformational change of PBL of a bacterial ProRS in the presence of adenylate analog. (adapted from ref 45).

Their work revealed that the strongly correlated and anticorrelated motions between the PBL and various segments of the protein were disrupted due to the deletion of the INS domain (Figure 5). Altered PBL dynamics and a decreased catalytic efficiency were also observed due to mutations at the domain–domain interface of Ec ProRS.^50^ In this work, a thorough MD simulation study as well as experimental mutational and kinetic studies were conducted by undergraduate researchers majoring in Biochemistry/Molecular Biology and Chemistry.

**Figure 5:**
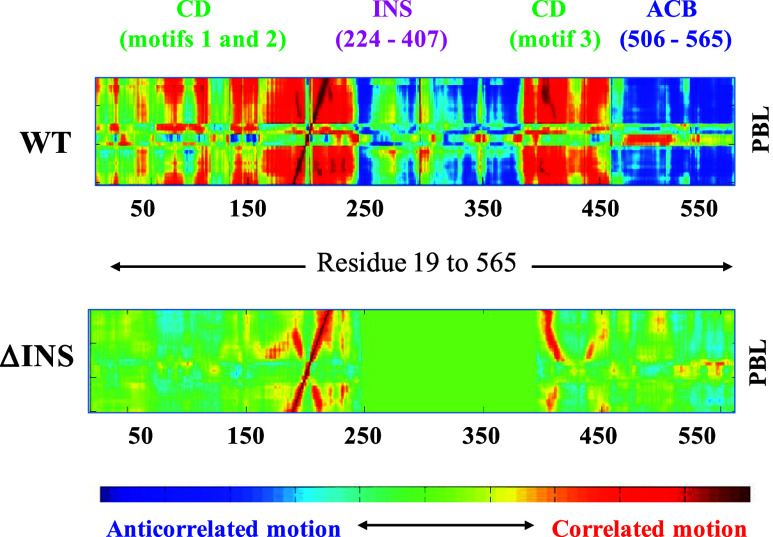
Dynamic cross-correlations between the C_a_ atoms of the PBL (residues 190-220) and residues 19-565 of the WT and the deletion mutant ΔINS (adapted from ref 45).

##### 3.1.2.3 Identification of Evolutionarily Conserved Inter-Domain Communication Pathways

Our focus then shifted to the identification of long-range communication pathways between distant domains in Ec ProRS (Figure 3). This was an excellent project for exposing students to various tools and techniques of biological science. A group of undergraduate collaborators involved in this project used site-directed mutagenesis, DNA sequencing, protein overexpression and purification, in vitro transcription, enzyme kinetics, whereas another set of students used statistical coupling analyses, MD simulations, and principal component analyses (PCA). Thus, using bioinformatics, MD simulations, and biochemical studies, four probable communication pathways between the catalytic and editing domains of Ec ProRS were identified through which signals flow that modulate PBL dynamics.^48^ These pathway residues are either conserved or coevolved residues, and through correlated motions, these residues facilitate the coupled dynamics between functional sites of Ec ProRS.

The highlight of this work is the automation of the method to identify evolutionarily conserved and coevolved residues that mediate long-range communications in enzymes that employ allostery. Earlier, we employed combined molecular simulations and statistical coupling analysis (SCA, a method to analyze evolutionarily coupled and conserved residues in a given protein family) to identify residue interactions that could mediate coupled-domain dynamics in Tt LeuRS.^47^ Later, the protocol was automated by an undergraduate, and the resultant Statistical Thermal Coupling Analysis (STCA) method was used to investigate the molecular mechanism by which the PBL dynamics is modulated by distant protein segments of Ec ProRS. The STCA method employs Dijkstra’s algorithm to trace interaction networks between functional sites from a pool of thermally and evolutionarily coupled residues that are identified using SCA and molecular simulation data. Multiple pathways of residue–residue interactions were identified between the catalytic and INS domains that could modulate the catalytically important PBL dynamics of Ec ProRS.^48^ Experimental (alanine-scanning mutagenesis and kinetic studies) and computational (in silico mutation and thermal fluctuations analysis) studies were conducted to examine the effects of mutations of “on-pathway” residues on enzyme function. The STCA method was used to identify evolutionarily optimized intrinsic conformational dynamics and residue networks that could mediate functional dynamics in other AARSs.^51^

##### 3.1.2.4 Intrinsic Dynamics and Catalytic Function of AARSs

Through two course-embedded research projects conducted in different years of the biophysical chemistry course, students investigated how intrinsic protein dynamics influence the enzymatic function of AARSs. In 2012, a cohort of 20 students performed normal-mode analysis on 20 AARSs and successfully classified them based on intrinsic dynamic properties.^52^ Their results suggested that dynamic-based classification could be a valuable approach for functional annotation of proteins. One of our research students also contributed to a follow-up study that addressed reviewer comments from this work. In a separate project conducted in 2019, 12 biophysical chemistry students focused on protein modeling and simulations, while three research students from our group carried out experimental mutagenesis and kinetic assays. Together, they demonstrated that residues distal to the catalytic active site play a significant role in prolyl-adenylate formation (eq 1) catalyzed by ProRS, primarily by maintaining intrinsic protein flexibility.^53^

##### 3.1.2.5 Preorganizing Dynamics by Noncatalytic Domain

Using QM/MM simulations, we developed a dynamic model of adenyl group transfer during prolyl-adenylate formation (eq 1), enabling us to investigate the role of the insertion (editing) domain in catalysis. Several enzyme variants were constructed, including the ΔINS deletion mutant and multiple active site point mutations. The energy profiles for adenylate formation were computed for both the wild-type enzyme and the mutants. These simulations provided activation barriers and reaction free energies for each case. Experimental site-directed mutagenesis confirmed the loss of enzymatic activity in the mutants. To quantify catalytic efficiency, we applied Marcus model,^54^ which revealed that deletion of the INS domain resulted in approximately a 50% reduction of the catalytic power, highlighting the critical role of the editing domain in conformational preorganization. Compared to water, the enzyme matrix increased the catalytic rate by 10^8^-fold (Figure 6). Moreover, the study identified two distinct dynamic contributions to catalysis: (1) motions intrinsic to the INS domain that facilitate conformational preorganization essential for catalysis, and (2) electrostatic rearrangements within the active site that reshape the energy landscape and optimize substrate orientation for productive collisions. These findings underscore how distal domain motions can preorganize the active site to enhance enzymatic performance.^55^

In this project, six undergraduate students participated, half were involved in conducting experimental studies, while the other half focused on QM/MM calculations and simulations. This collaborative effort provided a valuable opportunity for students to experience how complementary experimental and computational techniques can be integrated to deepen our understanding of enzymatic mechanisms at the molecular level.

##### 3.1.2.6 Molecular Recognition – Artificial Intelligence in Drug Discovery

Machine learning has achieved remarkable success in predicting key molecular properties, including the energy gap between the highest occupied molecular orbital (HOMO) and the lowest unoccupied molecular orbital (LUMO), 3D protein structures, and protein–small molecule (inhibitor) interactions. In our lab, in collaboration with undergraduate students majoring in computer science and chemistry, we have developed a deep learning tool based on a graph convolutional neural network (GCN) to predict molecular properties and binding affinities.^56^ This method integrates molecular graph convolution with an artificial neural network to extract substructural features relevant to properties such as binding affinity and frontier orbital energies.^57^ Initial studies have shown that the model can identify molecular substructures rooted in quantum chemical principles and capture the physical interactions between molecules and their active site environments. As part of an ongoing project, we are exploring the role of dynamics in the molecular recognition of ProRS, intending to identify species-specific inhibitors. Overall, the integration of task-based AI applications into chemistry research^56,57^ is providing our students with valuable opportunities to engage with cutting-edge technologies and stay current with advancements in artificial intelligence.

### 3.2 Impact of Crowding and Confinement on Dynamics

Another major factor that influence protein function and often evades consideration is the crowded cytoplasm, where the concentration of macromolecules such as proteins, nucleic acids, ribosomes, and lipids ranges between 100 and 450 g/L.^58^ This environment, entirely different from the dilute condition used in *in vitro* studies, can cause crowding and confinement effects.^59–61^ The crowding is inherently a dynamic phenomenon both in terms of the diffusional aspect of the crowders and the conformational dynamics of the enzyme. The preferential exclusion or accumulation of cosolutes (crowders) from/on the protein surface, proposed by Timasheff^62^ in the early 80s, is considered to be the core thermodynamic mechanism behind the crowding effect. Thus, the effects of macromolecular crowding on protein structure and dynamics can be explained based on the excluded volume effect and soft interactions; the former is entropic, and the latter is enthalpic by nature. The interplay of enthalpic-entropic changes stemming from solvent-cosolute and cosolute-macromolecule interactions has only been partially revealed.

As mentioned earlier, AARSs undergo large-scale conformational transition upon substrate binding. Conformational redistribution/transition upon substrate binding involves reorganization of noncovalent interactions among protein side-chains, as well as with the surrounding molecules. It has been observed that molecular crowding has a notable effect on protein stability and is known to significantly influence binding affinity and catalysis. Effects of molecular crowding on the conformational dynamics of multidomain AARSs, especially the transition rate from ″open″ to ″closed″ conformation and sampling of alternative conformations during substrate binding are expected to be influenced by crowding agents. Similarly, the energetics associated with substrate binding and catalysis in these enzymes are likely to be impacted by molecular crowding. Therefore, to accurately model the interplay of dynamics and molecular recognition/catalysis by AARSs, the impact of molecular crowding on conformational dynamics and catalytic functions were thoroughly examined with undergraduate researchers, employing experiments, computations, and spectroscopy, and using various synthetic crowders of different shape, size and chemical properties.

**Figure 6:**
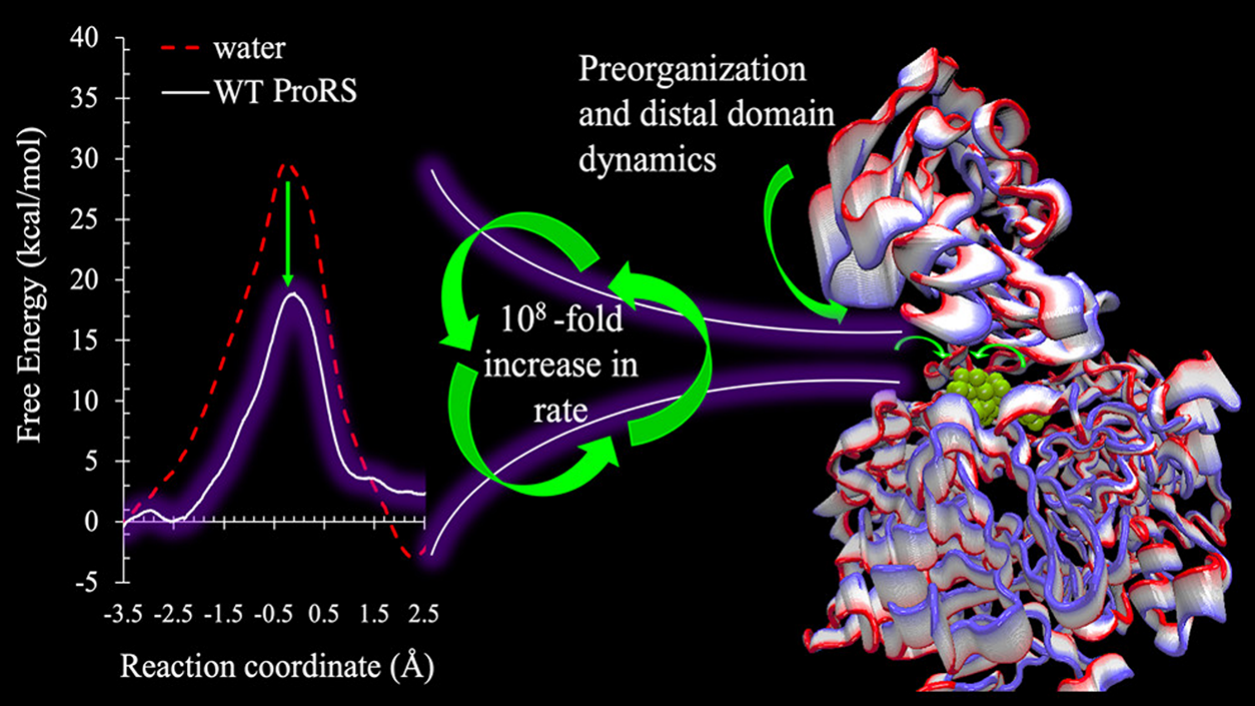
Loss of catalytic power of Ec ProRS due to the deletion of INS domain, as expressed in the form of decrease in catalytic rate (adapted from ref 55).

#### 3.2.1 Small-Molecule Crowders

The effect of molecular crowding by small crowders/cosolutes, such as dextrose and sucrose on the structure and function of Ec ProRS was investigated. To gain insight into the mechanistic details of the crowding effect, kinetic studies were conducted. In parallel, spectroscopic and quantum chemical studies were employed to probe the “soft interactions” between crowders and protein side chains. Additionally, the dynamics of the dimeric protein was examined in the presence of crowders using long-duration classical MD simulations. The results of this study revealed a shift in the conformational ensemble, which is consistent with the preferential exclusion of cosolutes. The “soft interactions” model of the crowding effect also explained the alteration in kinetic parameters. Thus, crowding by small-molecule agents alters the conformational dynamics of Ec ProRS, ultimately affecting its catalytic function.^60^

#### 3.2.2 Polyethylene Glycol (PEG) Crowders

A recent study with PEG crowders revealed decreased catalytic activity of Ec ProRS, where the smaller molecular weight PEGs had the maximum impact. The molecular mechanism of the crowding effects of PEGs is not clearly understood. PEG may impact protein conformation and dynamics, thus its function. Once again, with the help of several undergraduates, the effects of PEG molecules of various molecular weights and concentrations on the conformation and dynamics of Ec ProRS were investigated using a combined experimental and computational approach including intrinsic tryptophan fluorescence spectroscopy, atomic force microscopy, and atomistic MD simulations. Results of the study suggest that lower molecular weight PEGs in the dilute regime have modest effects on the conformational dynamics of Ec ProRS but impact the catalytic function primarily via the excluded volume effect; they form large clusters blocking the active site pocket. In contrast, the higher molecular weight PEGs in dilute to semidilute regimes have a significant impact on the protein’s conformational dynamics; they wrap on the protein surface through noncovalent interactions. Thus, lower-molecular-weight PEG molecules impact protein dynamics and function via crowding, whereas larger PEGs induce confinement effects.^61^

Additionally, at elevated concentrations, Ec ProRS exhibits liquid–liquid phase separation and forms condensates in the presence of 10–20% high-molecular-weight PEG. The molecular mechanism underlying this condensation is being investigated using protein labeling, confocal microscopy, and complementary experimental and computational techniques. These crowding studies affirmed that large and small crowders/cosolutes have considerable impacts on the structure, dynamics, and function of modular proteins. These results have implications for the development of inhibitors for protein targets in a crowded cellular environment and for stabilizing protein-based pharmaceuticals and industrial enzymes.^60^

#### 3.2.3 Crowder-induced Fluorescence Quenching of Tryptophan

Another physical science project involving spectroscopy and computational study was assigned to undergraduate students majoring in pure chemistry and biochemistry/molecular biology. To study protein conformational changes in crowded environments, we routinely use intrinsic tryptophan fluorescence spectroscopy. To better understand how the fluorescence properties of free tryptophan are influenced by synthetic crowding agents, such as dextran, ficoll, PEGs, polyvinylpyrrolidone, and their respective monomers, which are commonly used to mimic intracellular crowding, a comprehensive study was conducted in collaboration with eight undergraduate students. Half of the students carried on experiments, while the remaining half conducted computations. The results provided novel physical insights into the quenching mechanisms (Figure 7) of these widely used synthetic crowders. Their study demonstrated that the molecular mechanisms of quenching differ between polymeric and monomeric crowders. Using Stern–Volmer plots and temperature-dependent analyses, the quenching of free tryptophan was shown to involve static, dynamic, and sphere-of-action mechanisms (Figure 7). In parallel, computational studies employing Kohn–Sham density functional theory offered deeper insight into the role of intermolecular interactions and solvation, revealing distinct quenching behaviors among the different crowders.^63^

**Figure 7:**
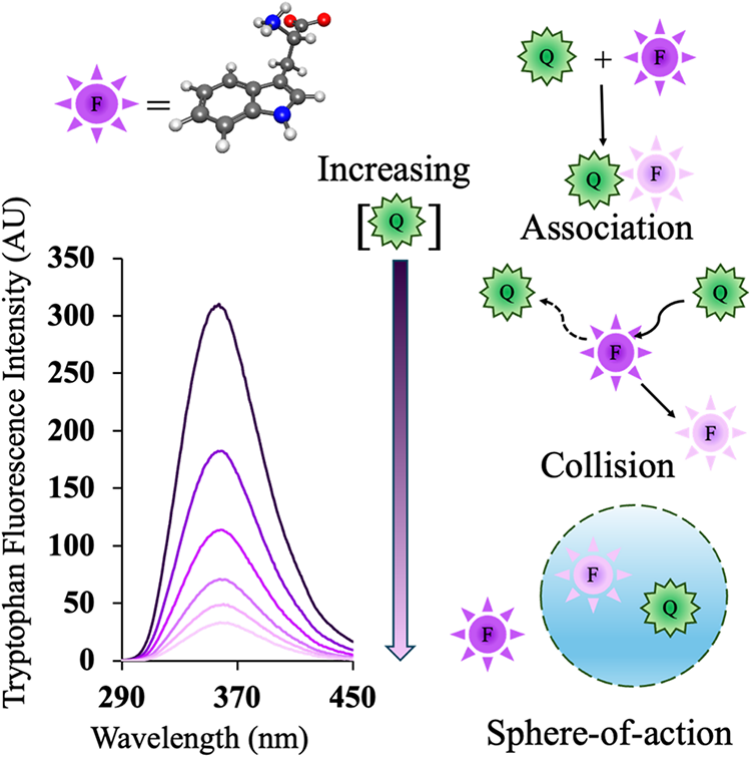
Molecular mechanisms of tryptophan fluorescence quenching due to monomer and polymer crowders (adapted from ref 63, which is licensed under CC-BY 4.0).

#### 3.2.4 Polyethylene Glycol Structure, Impurity, and Fluorescence

While studying the effects of molecular crowding on protein structure, dynamics, and function, we encountered an intriguing challenge related to the unexpected fluorescence exhibited by PEG with a molecular weight of 20,000 g/mol (PEG 20k). PEGs, polymers of ethylene glycol of varying sizes, are widely used in biological research and medicine due to their reputation as biologically inert compounds. Given their lack of a contiguous π-system, PEGs are not expected to exhibit fluorescence. However, recent studies have reported fluorescence in such nontraditional fluorophores. To investigate whether PEG 20k fluoresces, a team of undergraduate students conducted a comprehensive study using both experimental and computational approaches. Their findings revealed that although PEG 20k can exhibit “through-space” delocalization of lone electron pairs in aggregates or clusters formed via intra- and intermolecular interactions, the actual source of fluorescence between 300 and 400 nm is the stabilizer molecule, 3-*tert*-butyl-4-hydroxyanisole, present in commercially available PEG 20k. This discovery represents a significant achievement by undergraduate researchers and reinforces the idea that undergraduates can meaningfully contribute to solving complex research problems. Their paper^64^ has been well-cited by others in the field.

#### 3.2.5 Conformational Fluidity of Intrinsically Disordered Protein in the Crowded Environment

A MD simulation-based study was carried out on two intrinsically unstructured proteins versus two folded proteins (as controls) in the presence and absence of molecular crowders. The study revealed that polymeric ethylene glycol crowders stabilize the disordered proteins through enthalpic as well as entropic effects that are significantly more than their monomeric counterpart. Additionally, the study exhibited how molecular crowders induce a significantly diverse ensemble of dynamic scaffolds needed to carry out diverse functions.

Students working on this project learned the concepts of intrinsically disordered proteins and their thermodynamic behavior related to their functions. The students gained experience in carrying out atomistic simulations using a high-performance computing cluster. In addition, they performed a details analysis of energetics to compute enthalpic versus entropic contributions of crowding. This project was embedded in a biophysical chemistry course; a total of nine students of the class along with a research students coauthored the article.^65^

### 3.3 Conformational Changes in the Spike Protein and Oxidative Stress

Over 7 million people died due to the coronavirus disease (COVID-19), which started in late 2019. It is considered to be the most significant public health crisis in modern history. At the onset of the pandemic, our university went into lockdown around mid-March and later reopened for the fall semester with strict safety protocols, including physical distancing, reduced class sizes, and rotating student attendance. Despite these limitations, we initiated research to explore the scientific basis behind the varying severity of COVID-19 among patients. Our focus centered on oxidative stress, the redox chemistry of the thiol–disulfide equilibrium, and the role of the viral spike protein. We selected computational and literature review projects that could be conducted remotely, and we held regular research meetings via Microsoft Teams.

#### 3.3.1 Oxidative Stress and Covid-19 Severity

In the summer of 2020, during the COVID-19 lockdown, our team, comprising nine undergraduate research students, conducted a literature review on the role of oxidative stress in severe acute respiratory syndrome coronavirus SARS-CoV (SARS) and severe acute respiratory syndrome coronavirus SARS-CoV-2 (COVID-19) infections. This topic was selected because students had recently studied reactive oxygen species (ROS) and antioxidants in the context of cellular respiration, learning how their imbalance leads to oxidative stress. Working in small subgroups, the students reviewed the literature and summarized their findings with accurate citations. The resulting review paper was published in *The Protein Journal* and has since received over 300 citations.^66^

#### 3.3.2 Redox Chemistry and the Impact of Thiol–disulfide Balance

It is known that the receptor-binding domain has four disulfide bonds, which are exposed to the surface. The thiol to disulfide oxidation is thermodynamically favorable, and during oxidative stress, proteins’ cysteine thiol groups oxidize to disulfides.^67^ Structural studies on SARS-CoV and SARS-CoV-2 spike protein demonstrated that the receptor binding domain (RBD) forms a stable complex with the cell surface receptor angiotensin converting enzyme 2 (ACE2). As a part of the renin angiotensin system, ACE2 regulates the conversion of angiotensin II – a known vasoconstrictor and enhancer of oxidative stress. Thus, using MD simulation, the RBD-ACE2 complex was probed with and without disulfides on these proteins. The binding affinity calculations demonstrate an increased binding when disulfides are present. The study demonstrated that significant destabilization of the ACE2-RBD complex occurred upon reduction; the conformation of RBD altered due to the loss of disulfides, resulting in fewer interactions at the protein–protein interface. The work was followed up by a comprehensive MD simulation and probing the conformational changes of the receptor binding motif due to reduction of all disulfides into thiols (Figure 8). It was observed that the disruption of the disulfide bond causes a significant change in the receptor binding motif, and computation of binding affinity reaffirms that a docking-ready conformation is produced by the elevated oxidative stress in our body.^68^

**Figure 8:**
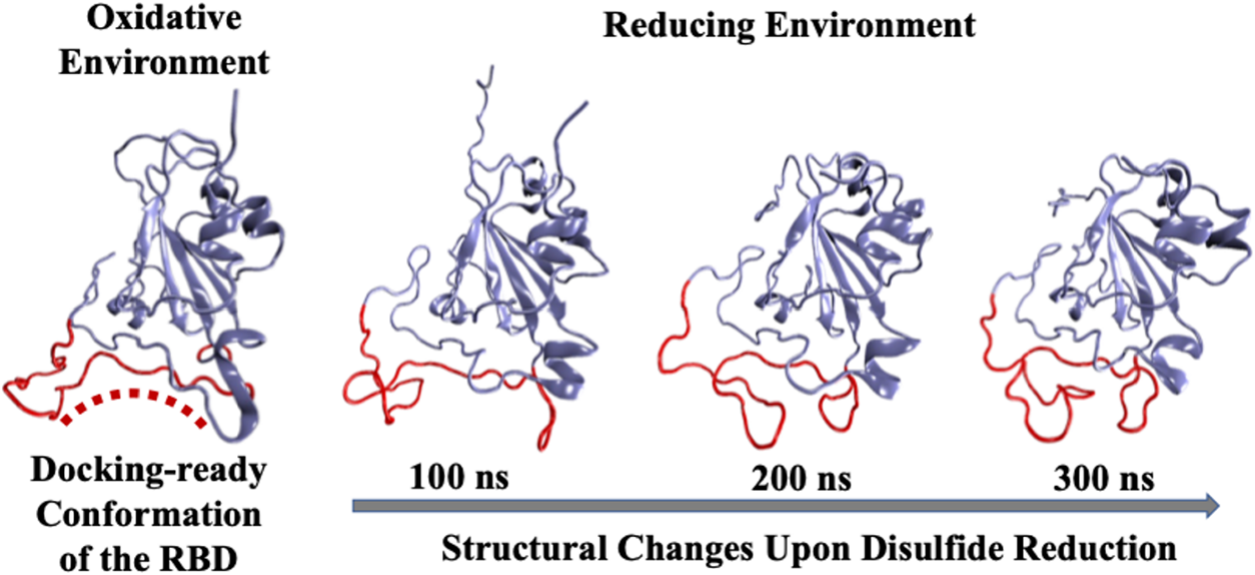
Structural changes of the receptor-binding motif upon reduction of the disulfides to thiolates (adapted from ref 68, which is licensed under CC-BY-NC-ND 4.0).

Students working in this project developed skills in constructing solvated atomistic models of proteins, conducting MD simulations, and performing collective dynamics analyses. They also learned to calculate polar solvation energies and protein–protein binding affinities using conformations extracted from MD trajectories.

#### 3.3.3 Variants of SARS-CoV-2 and Binding to ACE2 Receptor

The following year, as part of a course-embedded project in a biophysical chemistry course, students conducted short-time scale MD simulations to evaluate the binding strength of various SARS-CoV-2 spike protein variants with ACE2 receptors. The study offered valuable molecular-level insights into how the enhanced transmissibility and infectivity of the Delta and Omicron variants can be attributed to a small number of interacting residues at the binding interface. These findings have potential applications in the development of future antibody constructs and structure-based antiviral drug design.^69^ This project provided a significant synergistic learning experience by enabling biophysical chemistry students to develop computational skills through the exploration of a real-world problem. All 23 students in the class coauthored the resulting article.

#### 3.3.4 Vitamin D and the Severity of COVID-19 Infection

As mentioned earlier, the interaction between the SARS-CoV-2 spike protein and the human ACE2 receptor, both of which contain multiple cysteine residues, is influenced by the disulfide–thiol balance within the host cell. This redox status is affected by oxidative stress, which arises from an imbalance between reactive oxygen/nitrogen species and antioxidants. Previous studies have shown that vitamin D supplementation may reduce oxidative stress, and it has been proposed that vitamin D, at physiological concentrations, can have preventive effects against various viral infections, including COVID-19. To explore the molecular-level interplay between vitamin D deficiency, oxidative stress, and COVID-19 severity, a course-embedded review project was designed in spring of 2021 for the Biochemistry II course, which covers metabolic pathways. Through this literature-based project, 28 undergraduate students investigated the potential molecular mechanisms by which vitamin D could modulate host cell redox status and inhibit viral entry. They conducted a comprehensive literature review, analyzed the findings, and authored a paper highlighting the potential role of vitamin D in preventing COVID-19 infection or mitigating disease severity.^70^

### 3.4 Spectroscopic Tools for Disease Diagnostics

Undergraduate students are actively engaged in research projects, collaborating with the Mayo Clinic physicians to develop innovative methods for disease detection. This immersive model offers a unique opportunity to work side-by-side with both academic and clinical professionals, exposing students to real-world challenges. They experience firsthand how classroom knowledge translates into practical applications, while also learning advanced techniques to address urgent medical needs. Currently, a group of three to four students is engaged in developing a noninvasive spectroscopic method for cancer detection.

### 3.5 Research as a Tool to Teach Chemistry and Student Learning Outcomes

Research has traditionally been used as a pedagogical tool in chemistry education, offering students unique opportunities for engagement that cultivate critical thinking and problem-solving skills.

#### 3.5.1 Reinforcing Students’ Learning Through Connected Subdisciplines

Through a range of project-based experiences, students develop diverse techniques and tools (Table 1) to investigate the molecular mechanisms underlying enzymatic function, with particular emphasis on structure–dynamics–function relationships. These investigations reinforce core chemical principles across multiple subdisciplines while also exposing students to technical approaches spanning interconnected STEM fields, including quantum physics, molecular biology, bioinformatics, and computational science (Table 1, Figure 9). Within both experimental and computational laboratory settings, students explore key biological pathways and the proteins that mediate diverse chemical reactions. Experimental laboratories emphasize chemical handling, protein manipulation, biological assays, spectroscopic analyses, biomolecular imaging, and systematic data collection with an emphasis on quantifying uncertainties. In parallel, computational laboratories introduce theoretical frameworks, command-line operations, scripting, and high-performance computing workflows, including electronic structure calculations, classical and quasi-classical MD simulations, molecular docking, and neural network–based analyses (Table 1, Figure 9). Cross-training is encouraged, with students in computational cohorts gaining access to experimental tools and vice versa. Faculty-led group meetings further enrich the experience by fostering thoughtful discourse that bridges the two spheres of scientific practice and deepens interdisciplinary understanding.

**Figure 9:**
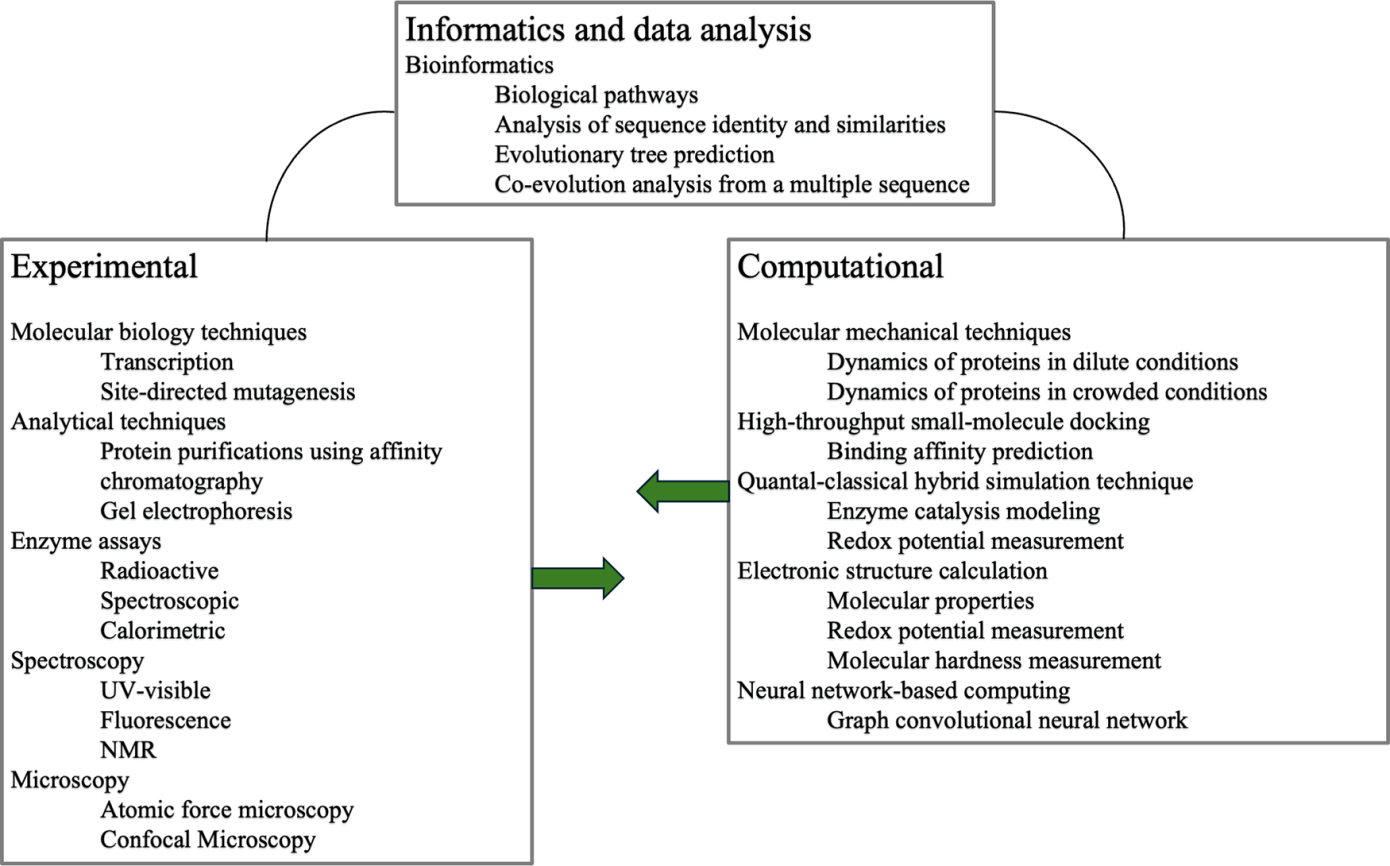
Techniques acquired by students through the combined experimental and computational lab.

#### 3.5.2 Students’ Learning Outcomes and Discovery-guided Projects

Protein dynamics is a central feature of enzymology. This is especially true for the Area 1 (Figure 1) project with AARSs, which are one of the fundamental enzymes involved in protein biosynthesis,^10,12^ and quinone reductases that are involved in redox chemistry. Understanding of these modular enzymes and their structure-dynamics-function relationships offers the core biophysical concepts and allows students to grasp topics such as the quantum nature of electrons and protons, polarization and hardness of a molecule, forces that help to fold biomolecules, molecular recognition, and catalytic functions (Table 2, column 1). Similarly, in Area 2, exploring how a protein’s function changes within a crowded cellular environment requires the creation of experimental conditions that mimic the intracellular milieu. Engaging with these projects provides students with foundational research experiences that span theoretical concepts, from electron-electron interactions to the conformational dynamics of macromolecules (Table 2, Column 1). Area 3 projects, focused on oxidative stress and COVID-19, offer students a novel platform for literature review and critical inquiry, enabling them to formulate questions that can contribute meaningfully to the scientific community. Area 4 projects empower students to apply classroom knowledge in solving real-world medical challenges.

**Table 2:** Student Learning Outcomes Achieved through Traditional Research Experiences

**areas of research and topics**	**student learning outcome**
**area 1: protein structure and functional dynamics**	
• molecular orbitals theory and molecular properties	
• intermolecular forces and molecular recognition	**knowledge**
• evolutionarily retained protein dynamics	• structure, bonding, and chemical properties
• coupled dynamics and substrate binding	• chemical and biochemical reactivities at the molecular level
• dynamics and hydride transfer: ping-pong kinetics	• assessment of errors in measurements
• dynamics and group transfer: prolyl-adenylate formation	
**area 2: crowding, confinement, and condensation**	
• molecular crowders: monomeric versus polymeric ones	
• fluorescence of tryptophan in crowded condition**s**	**skills**
• polyethylene glycol: structure and properties	• computational lab practices
• conformational dynamics and their changes	• experimental lab practices
• molecular mechanisms: crowding versus confinement	• literature review, and relating a topic to the current state of the knowledge
• biomolecular condensations	
**area 3: oxidative stress and conformational changes of spike protein**	
• oxidative stress, biomolecules that maintain thiol-disulfide balance	**responsibilities**
• impact of redox changes on spike protein binding	• safe laboratory practices
• effect of vitamin D on redox stress	• ethical and professional responsibilities
• impact of redox changes on conformational changes of the receptor binding motif of the spike protein	• communicating science to broader community
**area 4: spectroscopic tools for disease diagnostics**	
• functional groups, characteristic signal	
• standardizing methods	
• computational data analysis	

Student learning outcomes (Table 2, column 2) are evaluated after their time in the lab, based on completion of their research projects and using items such as research report and dissemination through student presentation at local, regional, and national conferences. Analysis of student learning outcomes demonstrates that the competencies cultivated through these projects fall into three key domains: knowledge, skills, and responsibilities (Table 2, Column 2). Students gained substantive knowledge in molecular structure and bonding, chemical reasoning, and error analysis. They also developed a wide range of laboratory and research skills, including experimental and computational techniques, literature review, scientific writing, and communication. Finally, students also became well-versed in responsible laboratory conduct, including safety protocols and ethical research practices. These immersive experiences fostered a strong sense of ownership and autonomy in their scientific work.

The learning outcome is further evidenced by the number of student-coauthored publications (Figure 10, Table 3). As previously described, the interdisciplinary nature of these projects immerse students in real-world scenarios that cultivate problem-solving skills informed by integrated knowledge from multiple STEM subdisciplines. Simultaneously, students cultivate essential skills: observing phenomena, critically analyzing data, interpreting the chemical significance of results, and synthesizing new knowledge—skills that shape them into thoughtful explorers of chemistry. Finally, they learn to articulate and communicate their insights through the writing of scientific articles. Moreover, such hands-on training has significantly supported students in pursuing advanced education and careers. Approximately 50% of our former research students have enrolled in prestigious Ph.D. programs, 20% have been accepted into medical and pharmacy schools, and many others have secured positions in biotechnology and pharmaceutical companies immediately after graduating from UWEC.

**Figure 10:**
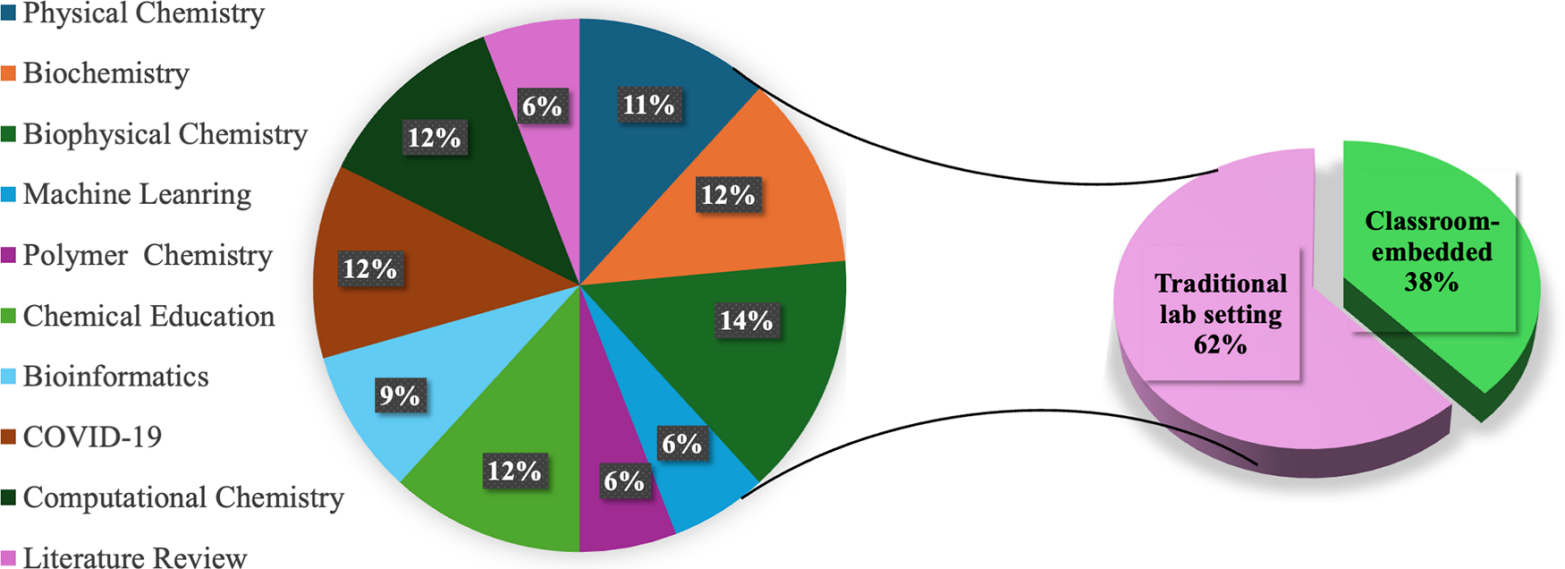
Peer-reviewed publications from the past two decades stem from both traditional research settings and course-embedded initiatives at UWEC. The pie chart illustrates the distribution of these publications across various chemistry subdisciplines as determined based on the primary focus of the research.

**Table 3:** List of Student Co-authored Publications Since 2009

**research model**	**number of undergraduates involved**	**number of peer-reviewed publications**	**number of student coauthors**
**traditional laboratory**	115	18	91^a^
**CURE**	220	10	147

a Multiple occurrences of a few students as coauthors.

#### 3.5.3 Limitations of Course-Embedded Research

Although the CURE model has been implemented in the Biophysical Chemistry lab for over a decade, it is not without limitations. Several key challenges and the strategies employed to address them are mentioned here. One challenge is maintaining student motivation, which occasionally falls short of the level required for a structured research project. Given the diversity of academic interests among students, project design must be aligned with topics that resonate with them. To enhance engagement, research themes were intentionally selected from the intersection of enzymology and public health. For instance, in the year following the COVID-19 pandemic, students investigated variants of the SPIKE protein. The following year, the focus shifted to the dynamics of intrinsically disordered proteins, which are implicated in many diseases. Additionally, the computational and data-intensive nature of these projects presents technical hurdles for some students. To mitigate this, we initially relied on web-based tools such as normal-mode analysis and molecular docking, allowing students to download results and perform visual analyses using interactive software like VMD. With the acquisition of a high-performance computing cluster, we transitioned to ready-to-use shell scripts. Students are now trained to use command-line interfaces, submit jobs, and retrieve results from remotely connected campus supercomputers. Another limitation arises from students’ expectations in discovery-driven projects, which often yield open-ended results. To address this, the course begins with a discussion on the nature of CURE and its role in fostering deep learning through research. Students are reminded that these projects may not produce definitive outcomes but are valuable for developing scientific thinking. Motivated students are offered the opportunity to continue their research through a one-credit independent study, potentially leading to peer-reviewed presentations and publications. Finally, implementing CURE demands significant additional effort from faculty in terms of planning, mentoring, and execution—much of which goes unrecognized. Faculty in administrative roles can play a crucial part in developing creative solutions to acknowledge and support the extra workload associated with successful CURE implementation.

## 4 Conclusions

Over the past two decades, undergraduate research in Chemistry (Biochemistry and Biophysical Chemistry) at UW–Eau Claire has served as a powerful platform for training a large cohort of students. This program has provided exceptional opportunities for students to engage in real-world problem-solving, explore chemistry in depth, and develop the ability to describe the natural world through the lens of molecules, atoms, and electrons. Students have also gained mastery in subdisciplines of their choice. By investigating fundamental enzymological questions related to protein dynamics, students have developed significant insights into the roles of molecular motion in small molecule recognition and catalysis, biochemical processes under crowded conditions, and redox stress-induced conformational changes. In addressing core questions, such as why proteins are large despite catalytic chemistry occurring within a relatively small active-site pocket, they acquired a wide range of laboratory skills. In addition, students honed their abilities in literature review, scientific writing, and communication. More importantly, being involved in authentic research, they made significant contributions to the field of enzymology and expanded our understanding of the structure–dynamics–function relationship in proteins. Last but not least, through these intregative learning experiences, they emerged as ethically responsible stewards of science, future educators, lifelong learners, and curious explorers dedicated to the pursuit of truth.
